# White matter and gray matter changes related to cognition in community populations

**DOI:** 10.3389/fnagi.2023.1065245

**Published:** 2023-03-14

**Authors:** Wen-Xin Li, Jing Yuan, Fei Han, Li-Xin Zhou, Jun Ni, Ming Yao, Shu-Yang Zhang, Zheng-Yu Jin, Li-Ying Cui, Fei-Fei Zhai, Yi-Cheng Zhu

**Affiliations:** ^1^Department of Neurology, State Key Laboratory of Complex Severe and Rare Diseases, Peking Union Medical College Hospital, Chinese Academy of Medical Science and Peking Union Medical College, Beijing, China; ^2^Department of Cardiology, State Key Laboratory of Complex Severe and Rare Diseases, Peking Union Medical College Hospital, Chinese Academy of Medical Science and Peking Union Medical College, Beijing, China; ^3^Department of Radiology, State Key Laboratory of Complex Severe and Rare Diseases, Peking Union Medical College Hospital, Chinese Academy of Medical Science and Peking Union Medical College, Beijing, China

**Keywords:** MRI, DTI, white matter, gray matter, cognitive function

## Abstract

**Objective:**

Further studies are needed to improve the understanding of the pathological process underlying cognitive impairments. The purpose of this study is to investigate the global and topographic changes of white matter integrity and cortical structure related to cognitive impairments in a community-based population.

**Methods:**

A cross-sectional analysis was performed based on 995 subjects (aged 56.8 ± 9.1 years, 34.8% males) from the Shunyi study, a community-dwelling cohort. Cognitive status was accessed by a series of neurocognitive tests including Mini-Mental State Examination (MMSE), Montreal Cognitive Assessment (MoCA), category Verbal Fluency Test (VFT), Digit Span Test (DST), and Trail Making Tests A and B (TMT-A and TMT-B). Structural and diffusional MRI data were acquired. White matter integrity was assessed using fractional anisotropy (FA), mean diffusivity (MD), and peak width of skeletonized mean diffusivity (PSMD). Cortical surface area, thickness, and volume were measured using Freesurfer. Probabilistic tractography was further conducted to track the white matter fibers connecting to the cortical regions related to cognition. General linear models were used to investigate the association between brain structure and cognition.

**Results:**

Global mean FA and MD were significantly associated with performances in VFT (FA, β 0.119, *p* < 0.001; MD, β −0.128, *p* < 0.001). Global cortical surface area, thickness, and volume were not related to cognitive scores. In tract-based spatial statistics analysis, disruptive white matter integrity was related to cognition impairment, mainly in visuomotor processing speed, semantic memory, and executive function (TMT-A and VFT), rather than verbal short-term memory and working memory (DST). In the whole brain vertex-wise analysis, surface area in the left orbitofrontal cortex, right posterior-dorsal part of the cingulate gyrus, and left central sulcus were positively associated with MMSE and MoCA scores, and the association were independent of the connecting white matter tract.

**Conclusion:**

Disrupted white matter integrity and regional cortical surface area were related to cognition in community-dwelling populations. The associations of cortical surface area and cognition were independent of the connecting white matter tract.

## 1. Introduction

Cognitive decline is a major issue in elderly populations (Jia et al., 2020). It reduces personal life quality and increases the demand for nursing and social care, which leads to various socioeconomic burdens (Livingston et al., 2017; The Lancet Neurology, 2018). There are increasing concerns regarding the brain structure basis of cognitive decline. Some studies demonstrate that cerebral cortical atrophy is a major predictor of cognitive decline both in degenerative diseases, such as Alzheimer’s disease (Poulakis et al., 2018; Nunez et al., 2020; Qing et al., 2021), and in vascular cognitive impairment, such as cerebral small vessel disease (CSVD; Jokinen et al., 2012; Righart et al., 2013). Recently, with advanced imaging techniques, such as diffuse tensor imaging (DTI), more and more evidences reveal that subcortical white matter lesions, including white matter hyperintensity (WMH) volume (Lampe et al., 2019), WM microstructure injury (Cremers et al., 2016; Power et al., 2019; Caunca et al., 2020), and disrupted network connectivity (Lawrence et al., 2014; Tuladhar et al., 2016) are independently related to impaired cognition, even in non-dementia community populations. These evidences imply that both gray matter and white matter play important roles in cognitive function. In non-dementia populations, white matter lesions may occur earlier and appear to be more sensitive to cognitive impairments.

However, cortical gray matter and subcortical white matter are not isolated, they are spatially and functionally connected. Cortical atrophy can be secondary to the disruption of connecting fibers in the subcortical white matter (Lambert et al., 2016; Kim et al., 2020). In reverse, cortical atrophy can also lead to Wallerian degeneration of white matter tract (McAleese et al., 2017). A longitudinal study found that white matter hyperintensity expansion and gray matter atrophy were strongly correlated in patients with small vessel disease (Lambert et al., 2016), but this association was not found in older community-dwelling subjects (Dickie et al., 2016). The Northern Manhattan Study recently demonstrated that white matter hyperintensity volume was related to worse processing speed, both directly and indirectly through its effect on regional cortical thickness (Caunca et al., 2020). Lots of studies, including our previous work (Wang et al., 2021), tried to disentangle the relationship between cortical and subcortical brain structure with cognition (Righart et al., 2013; van Uden et al., 2015; Cremers et al., 2016; Dickie et al., 2016; Lambert et al., 2016; Vibha et al., 2018; Power et al., 2019; Celle et al., 2021; Rizvi et al., 2021). However, some of these studies only chose global gray matter or white matter parameters, such as total or regional cortical volume (Vibha et al., 2018), white matter hyperintensity volume (Celle et al., 2021), mean or tract-specific fractional anisotropy (FA) or mean diffusivity (MD; van Uden et al., 2015; Cremers et al., 2016; Power et al., 2019). Our previous work (Wang et al., 2021) also only investigated the relationship of imaging markers of CSVD and brain volume with cognition in the whole brain level. Results of the article showed no relationship between global gray matter parameters with cognition. Global parameters contained in former studies (van Uden et al., 2015; Cremers et al., 2016; Vibha et al., 2018; Power et al., 2019; Celle et al., 2021; Wang et al., 2021) could not provide precise spatial information in cerebral changes. Also, early cerebral changes may happen locally, which could not be detected in global level. Therefore, precise evaluation and mapping of white matter microstructure injury and cortical morphology would be expected to provide further information about the underlying pathological process of cognitive impairment.

In a population-based sample, we investigated the relationship between global and topographic changes in white matter and cortical structures and cognitive function. Furthermore, we determined whether the association between cortical changes and cognitive impairment was influenced by its connecting white matter tract.

## 2. Materials and methods

### 2.1. Participants

This cross-sectional analysis was based on the Shunyi study, an ongoing prospective population-based cohort study in community-dwelling adults. Between June 2013 and April 2017, we recruited 1,586 participants aged 35 years or older from five villages of Shunyi, a suburb of Beijing. Among these participants, brain magnetic resonance imaging (MRI) was completed in 1,257 subjects. We obtained 1,145 scans with adequate quality for structure segmentation. Cognitive tests (at least Mini-Mental State Examination and Montreal Cognitive Assessment scores) were applied to 1,069 participants. A total of 74 participants with previous stroke or dementia were excluded, leaving 995 participants in these analyses (Supplementary Figure 1). The study was approved by the ethics committee at Peking Union Medical College Hospital (reference No. B-160) and all participants had signed written informed consent.

### 2.2. Cognitive measurements

The cognitive tests included the Chinese version of Mini-Mental State Examination (MMSE; Zhang et al., 1999), Montreal Cognitive Assessment (MoCA; Wen et al., 2008), category Verbal Fluency Test (VFT), Digit Span Test (DST), and Trail Making Tests A and B (TMT-A and TMT-B; Zhang et al., 1998; Han et al., 2020). MMSE and MoCA were used to assess global cognition. In category verbal fluency test (VFT), participants were asked to verbally list as many animals/vegetables/fruits in 60 s. We counted up the total number of the three tests as the VFT score. The VFT was a measure of semantic memory, language and executive function. In the DST, the participants repeated the numbers forward and backward by increasing the series of numbers until the answers were incorrect. The forward and backward scores were summed. The DST was used to evaluate verbal short-term memory by the forward task and working memory by the backward task. We also used a digital TMT-A and TMT-B to assess the executive function, which was based on our mental status detection system with a Wacom template (patent NO. CN 103956171 B). Trail Making Test-Part B was modified into a Chinese version starting the trail with Chinese numeral “one” to the Arabic numeral “1,” Chinese numeral “two” to the Arabic numeral “2,” till Chinese numeral “twelve” to the Arabic numeral “12.” We extracted the completion time of TMT-A and TMT-B in this study.

When participants had MMSE scores ≤ 24 or cognitive complaints, they were asked to complete the Clinical Dementia Rating Scale (Morris, 1993). A dementia specialist made the dementia diagnosis by reviewing the clinical information, comprehensive neuropsychological (NP) assessments test results (e.g., abnormal NP test score or CDR ≥ 1), and functional evaluation (Han et al., 2020). The diagnosis of dementia needs to satisfy the 2011 NIA-AA Criteria for all-cause dementia (McKhann et al., 2011). A total of 27 participants with dementia were excluded from this analysis.

### 2.3. ApoE genotyping

Genomic DNA extracted from whole blood samples were amplified by PCR and sequenced with Sanger sequencing. ApoE genotype was identified by manually combining the alleles from the single nucleotide polymorphisms NM_000041.2:c.388 T > C (p.Cys130Arg; rs429358) and c.526C > T (p.Arg176Cys; rs7414) as follows: at nucleotides 388 and 526 (amino acids 130 and 176), ε2 = TT (CysCys), ε3 = TC (CysArg), and ε4 = CC (ArgArg). APOE ε4+ was identified as ε2/ε4, ε3/ε4, and ε4/ε4, and APOE ε4− was identified as ε2/ε2, ε2/ε3, and ε3/ε3 (Drogos et al., 2016; Su et al., 2019).

### 2.4. MR imaging

Details of MRI acquisition were described in our previous publications (Han et al., 2020). Relevant to this study, a T1 weighted (3D magnetization-prepared rapid gradient-echo sequence with 1 mm × 1mm × 1.3 mm resolution), a T2 weighted fluid-attenuated inversion recovery (FLAIR) sequence (1 mm × 1 mm × 5 mm resolution), a single-shell diffusion tensor image (DTI) with 30 directions at b = 1,000 s/mm^2^ and a non-diffusion weighted image with b = 0 s/mm^2^ (2.2 mm × 2.2 mm × 2.2 mm resolution, repeat twice) were used. We processed the imaging data using United Kingdom Biobank processing pipeline (Alfaro-Almagro et al., 2018)^1^ modified according to our MRI parameters.

Raw images with correct imaging parameters were visually inspected in the quality check process. Scans with artifacts including spikes, severe ghosting, severe head-motion and eye spillover, electromagnetic interference/perturbation which affected imaging of the brain were excluded from further analysis. Participants with congenital dysplasia, severe non-vascular brain structure damage such as hydrocephalus, space occupying lesions, significant infarctions were also excluded.

### 2.5. Cortical parcellation and hippocampus segmentation

The T1 weighted images were processed for cortical reconstruction (Dale and Sereno, 1993; Dale et al., 1999) and volumetric segmentation using the FreeSurfer (v7.2) software package.^2^ Three vertex-wise imaging measures, cortical thickness, cortical volume, and cortical surface area, were included to obtain a comprehensive description of cognition-related differences in cortical morphology. Before statistical analysis, the surface-based data were smoothed using a Gaussian kernel with the full-width half-maximum of 10 mm to increase the signal-to-noise ratio and reduce the impact of misregistration. Hippocampus volume was acquired from the recon-all pipeline. The estimated total intracranial volume was used to correct the brain size.

Freesurfer-processed MRI scans were visually examined based on the quality checking protocol of Enhancing Neuro ImaGing through Meta-Analysis (ENIGMA).^3^ Cortical segmentation quality, cortical labels and anatomical boundaries of each scan were checked and rated as 1 = “Good,” 2 = “Moderate,” 3 = “Fail” based on the severity of misclassification defined by the ENIGMA Cortical Control Guide 2.0. Scans graded as “Fail” were excluded from further analysis. Misclassifications in rest of the scans were manually corrected or deleted according to the troubleshooting guide from the FreeSurfer team.^4^ Scans with outliers (>3SD) were re-checked to examine segmentation quality.

### 2.6. White matter hyperintensity segmentation

WMH segmentation was carried out using the Brain Intensity Abnormality Classification Algorithm (BIANCA; Griffanti et al., 2016), which is a fully automated method for classifying voxels based on relative intensity and spatial features. White matter hyperintensity was manually labeled on FLAIR sequence in 25 participants as training data. The BIANCA output is a probability map that is thresholded at 0.95 to produce a binary map of lesions. The Dice coefficient was 0.87 in our dataset. Obtained white matter hyperintensity volumes were log-transformed because of their skewed distribution.

### 2.7. DTI processing

The original DTI data were corrected for eddy currents, head motion, and gradient distortion. The output was fed into the DTI fitting tool (DTIFIT, part of FSL), creating FA and MD maps. The global mean FA and MD were calculated by averaging the diffusion metrics within study-specific WM mask (the mean FA map thresholded at 0.2). The FA and MD maps were then processed using tract-based spatial statistics (TBSS) pipeline (Smith et al., 2006). This involves nonlinear registration of the FA images to a standard-space white matter skeleton. The resulting standard-space warp is applied to the MD map. Then, voxel-wise analysis of the association between cognition and DTI measures were conducted on these skeletons *via* FSL Randomize tool using general linear model. The DTI datasets was processed using UK Biobank diffusion pipeline.^5^ Changes made according to our MRI parameters include: (a) we did not carry out TOPUP in the study, because our diffusion images were scanned only in AP direction. Even though we used bipolar diffusion scheme to reduce eddy current distortions and corrected eddy current in the pre-procession, we cannot exclude brain distortion especially in frontal and temporal regions. (b) our DTI are single-shell images (b = 1,000 s/mm^2^), therefore, we did not run “bb_select_dwi_vols” command for multi-shell images of UKB.

Apart from the imagine-derived phenotypes using United Kingdom Biobank pipeline, we also computed the peak width of skeletonized mean diffusivity (PSMD) using psmd.sh,^6^ which has been considered as a novel imaging marker for small vessel disease based on skeletonization of white matter tract (Baykara et al., 2016).

### 2.8. Probabilistic tractography

In order to track the white matter fibers connecting to the cortical regions related to cognition, we also conducted probabilistic tractography (Supplementary Figure 2). We extracted the cortical clusters related to cognition from vertex-wise analysis in Freesurfer data as the seeding regions. The transformation matrix from Freesurfer surface space to native DTI volume space was constructed *via* mri_surf2surf, mri_surf2vol, tkregister2, and FLIRT (FMRIB’s Linear Image Registration Tool). Tractography began with within-voxel modeling of multi-fiber tract orientation structure *via* the bedpostx tool, and was followed by probabilistic tractography using probtractx2 (Behrens et al., 2007). Streamlines were seeded from each voxel in the cluster, and in order to tract all possible streamlines, we did not set the exclusion mask and termination mask. The default 0.5 mm step length, 5,000 samples and 2,000 steps were used. To avoid artifactual loops, streamlines that loop back on themselves were discarded (loopcheck). For each subject, the tracked streamline was normalized by the total number of generated streamlines (“waytotal” number). A threshold of 1% was used to binarize the probabilistic tractography to avoid false-positive streamline (Tian et al., 2018). Finally, the mean FA and MD in the resulting tract, which was connected to the cortical cluster, were calculated in individual native DTI space.

### 2.9. Covariates

Covariates were selected based on prior literature. Age (years) and education (years) were modeled as continuous variables. ApoE ε4 carrying situation was dichotomized as yes or no. All analyses were adjusted for age, sex, years of education, and ApoE ε4 carriership. Analyses involving the cortical structure, hippocampus volume, and WMH volume, were further adjusted for the total intracranial volume.

### 2.10. Statistical analyses

The baseline characteristics were presented as mean (standard deviation), median (interquartile range), or frequency (percentage). White matter hyperintensity volume and completion time of TMT-A and TMT-B were log-transformed to correct a skewed distribution. The cognitive scores were standardized to z-scores based on the means and standard deviations in the analysis to make relative comparisons across cognitive domains and accommodate differences in test units and scales.

The relationships of global cortical and white matter measures with cognition were examined using multiple linear regression with brain measures as determinants and cognition scores as dependent variables after controlling for the before-mentioned covariates. A total of 8 brain structure parameters and 6 cognitive scores were included in the study. We corrected for multiple testing across all models by the false discovery rate (FDR) correction using the Benjamini-Hochberg method (Benjamini and Hochberg, 1995). Results at a *p*-value < 0.05 by FDR correction were considered statistically significant. Statistical analyses were conducted by SAS 9.4.

Voxel-wise analysis of DTI data was performed using TBSS to evaluate the topographical changes of white matter related to cognition. We used a linear regression model with age, sex, education year, and ApoE ε4 allele carriership as covariates. The number of permutation tests was set at 5000 for statistical inference, and significant thresholds were determined using a threshold-free cluster enhancement with a value of p of 0.05 to correct for multiple comparison (Smith and Nichols, 2009).

Vertex-wise analyses of Freesurfer data were conducted to assess the relations between cortical changes and cognition using Permutation Analysis of Linear Models (PALM).^7^ Cortical surface area and volume analyses were controlled for age, sex, education year, APOE-ε4 allele carriership, and total intracranial volume. Cortical thickness analyses were controlled for age, sex, education year and APOE-ε4 allele carriership. The number of permutation tests was set at 5,000 and significant thresholds were determined using a threshold-free cluster enhancement with a *p*-value of 0.05.

To examine whether the relationship between cortical regions and cognition was influenced by white matter tract connecting to these cortical regions, we further constructed regression models with cortical measures in the significant cluster as predictors and cognitive scores as outcomes, additionally adjusted for the mean FA or MD of white matter tracts connected to the significant cluster.

Moreover, the MMSE scores were relatively lower in our study even in the non-dementia participants as all participants are rural residents and most of these participants were at a low educational level. Sensitivity analyses were performed, excluding the participants with MMSE < 17 for illiterate, MMSE < 20 for individuals with 1–6 years of education, and MMSE < 24 for individuals with 7 or more years of education according to the suggested cut-off points for dementia screening in a Chinese population-based normative study (Li et al., 2016).

### 2.11. Data availability

Anonymized data will be shared by request from the qualified investigator for the sole purpose of replicating procedures and results presented in the article after ethics clearance and approval by all members of the project group.

## 3. Results

### 3.1. Characteristics of the population

A total of 995 participants were included in this study. The demographics, cognitive test results, and neuroimaging characteristics of the population are shown in Table 1. The mean age was 56.8 years (SD 9.1 years) and 34.8% were male. Most participants were at a low educational level and the mean education time was 6.7 years (ranging from 0 to 15 years). The mean MMSE and MoCA scores were 26.5 (SD 3.2) and 19.2 (SD 4.9), respectively. The median white matter hyperintensity volume was 2.9 × 10^3^ mm^3^ (IQR 1.8–5.1 mm^3^ × 10^3^ mm^3^).

**Table 1:** Characteristics of the study population (*n* = 995).

Variables	
Age at MRI, year	56.8 (9.1)
Male	346 (34.8%)
Education, year	6.7 (3.2)
ApoE ε4 carrier	143 (15.6%)
Cognition tests	
MMSE	26.5 (3.2)
MoCA	19.2 (4.9)
Verbal Fluency Test (*n* = 945)	37.7 (8.7)
Digit Span Test (*n* = 931)	10.5 (2.1)
Digit Span Test-Forward (*n* = 931)	6.9 (1.6)
Digit Span Test-Backward (*n* = 931)	3.6 (1.0)
TMT-A, s, median (IQR; *n* = 961)	58.6 (43.5, 85.3)
TMT-B, s, median (IQR; *n* = 921)	90.0 (63.4, 130.4)
**Brain structures**	
Cortical GM volume, mL	428.6 (39.2)
Cortical GM surface area, mL	194.5 (16.8)
Cortical GM thickness, mm	2.4 (0.1)
Hippocampus volume, mL	8.0 (0.8)
WMH volume, mL, median (IQR)	2.9 (1.9, 5.1)
Global FA	0.4 (0.02)
Global MD × 10^−3^ mm^2^/s	0.8 (0.04)
PSMD×10^−3^ mm^2^/s	0.2 (0.05)
Total intracranial volume, mL	1505.6 (155. 6)

MRI indicates magnetic resonance imaging; MMSE indicates Mini-Mental State Examination; MoCA indicates Montreal Cognitive Assessment; TMT-A indicates Trail Making Test part A; TMT-B indicates Trail Making Test part B; GM indicates gray matter; FA indicates fractional anisotropy; MD indicates mean diffusivity; PSMD indicates peak width of skeletonized mean diffusivity. Data are displayed as mean (SD) or *n* (%) unless specified otherwise. Number of missing data of cognitive tests: Verbal Fluent Test, 50; Digit Span Test, 64; TMT-A, 34; TMT-B, 74.

### 3.2. Effect of global white matter and cortical measures on cognition

Compared with global cortical morphology measures, white matter had a stronger association with cognition (Table 2). Before FDR correction, global mean FA, MD and PSMD were all associated with VFT (FA, β = 0.119, *p* < 0.001; MD, β = −0.128, *p* < 0.001; PSMD, β = −0.98, *p* = 0.009) and TMT-A (FA, β = −0.083, *p* = 0.014; MD, β = 0.089, *p* = 0.014; PSMD, β = 0.105, *p* = 0.003). The global mean FA was also associated with MoCA (β = 0.081, *p* = 0.010). PSMD was associated with MMSE (β = −0.074, *p* = 0.033) and MoCA (β = −0.071, *p* = 0.031).

**Table 2:** Association of cortical gray matter, hippocampus, and white matter measures with cognition.

	MMSE				MoCA				Verbal fluency test				Digit span test				TMT-A				TMT-B			
Variable	β	SE	*p*	FDR-p	β	SE	*p*	FDR-p	β	SE	*p*	FDR-p	β	SE	*p*	FDR-p	β	SE	*p*	FDR-p	β	SE	*p*	FDR-p
Cortical GM volume, mm^3,a^	0.087	0.052	0.097	0.271	0.069	0.050	0.163	0.337	0.023	0.057	0.691	0.829	0.030	0.058	0.601	0.740	−0.015	0.054	0.785	0.861	0.114	0.054	**0.036**	0.135
Cortical GM surface area, mm^2, a^	0.093	0.057	0.102	0.271	0.093	0.054	0.084	0.252	0.053	0.062	0.391	0.619	0.047	0.063	0.455	0.631	−0.008	0.058	0.885	0.904	0.091	0.059	0.125	0.293
Cortical GM thickness, mm^a^	0.010	0.030	0.749	0.861	−0.008	0.029	0.789	0.861	−0.022	0.033	0.499	0.631	−0.007	0.033	0.826	0.881	−0.001	0.031	0.982	0.982	0.046	0.032	0.146	0.319
Hippocampus volume, mm^3, a^	0.089	0.036	**0.014**	0.086	0.077	0.034	**0.027**	0.128	0.052	0.039	0.185	0.341	−0.027	0.040	0.498	0.631	−0.007	0.037	0.859	0.896	0.060	0.038	0.114	0.287
WMH volume, log^a^	−0.038	0.044	0.391	0.619	−0.080	0.042	0.057	0.183	−0.106	0.048	**0.027**	0.128	−0.094	0.049	0.054	0.183	0.063	0.045	0.169	0.337	−0.012	0.046	0.789	0.861
Global FA^b^	0.023	0.033	0.491	0.631	0.081	0.031	**0.010**	0.086	0.119	0.035	**<0.001**	**0.021**	0.055	0.036	0.128	0.293	−0.083	0.034	**0.014**	0.086	−0.029	0.034	0.400	0.619
Global MD × 10^−3^ mm^2^/s^b^	−0.048	0.036	0.175	0.337	−0.043	0.034	0.204	0.364	−0.128	0.038	**<0.001**	**0.021**	−0.028	0.039	0.477	0.631	0.089	0.036	**0.014**	0.086	−0.029	0.037	0.442	0.631
PSMD×10^−3^ mm^2^/s^b^	−0.074	0.034	**0.033**	0.130	−0.071	0.033	**0.031**	0.130	−0.098	0.037	**0.009**	0.086	−0.045	0.038	0.238	0.408	0.105	0.035	**0.003**	**0.047**	−0.026	0.036	0.475	0.631

Bold text indicates a statistically significant correlation with a p-value less than 0.05. MMSE indicates Mini-Mental State Examination; MoCA indicates Montreal Cognitive Assessment; TMT-A indicates Trail Making Test part A; TMT-B indicates Trail Making Test part B; GM indicates gray matter; FA indicates fractional anisotropy; MD indicates mean diffusivity; PSMD indicates peak width of skeletonized mean diffusivity. β indicates standardized regression coefficient; SE indicates standard error; FDR-p indicates false discovery rate adjusted *p*-value.

a Models adjusted for age, sex, education, ApoE ε4 carrier, and total intracranial volume.

b Models adjusted for age, sex, education, and ApoE ε4 carrier.

After FDR correction, FA and MD were still significantly associated with VFT (FA, FDR-*p* = 0.021; MD, FDR-p = 0.021), and PSMD was marginally associated with TMT-A (FDR-*p* = 0.047). Other associations did not survive FDR correction.

For cortical measures, no significant associations were observed between global cortical GM volume, surface area, thickness and cognitive scores after FDR correction.

### 3.3. Disruptive WM integrity related to cognition

**Figure 1:**
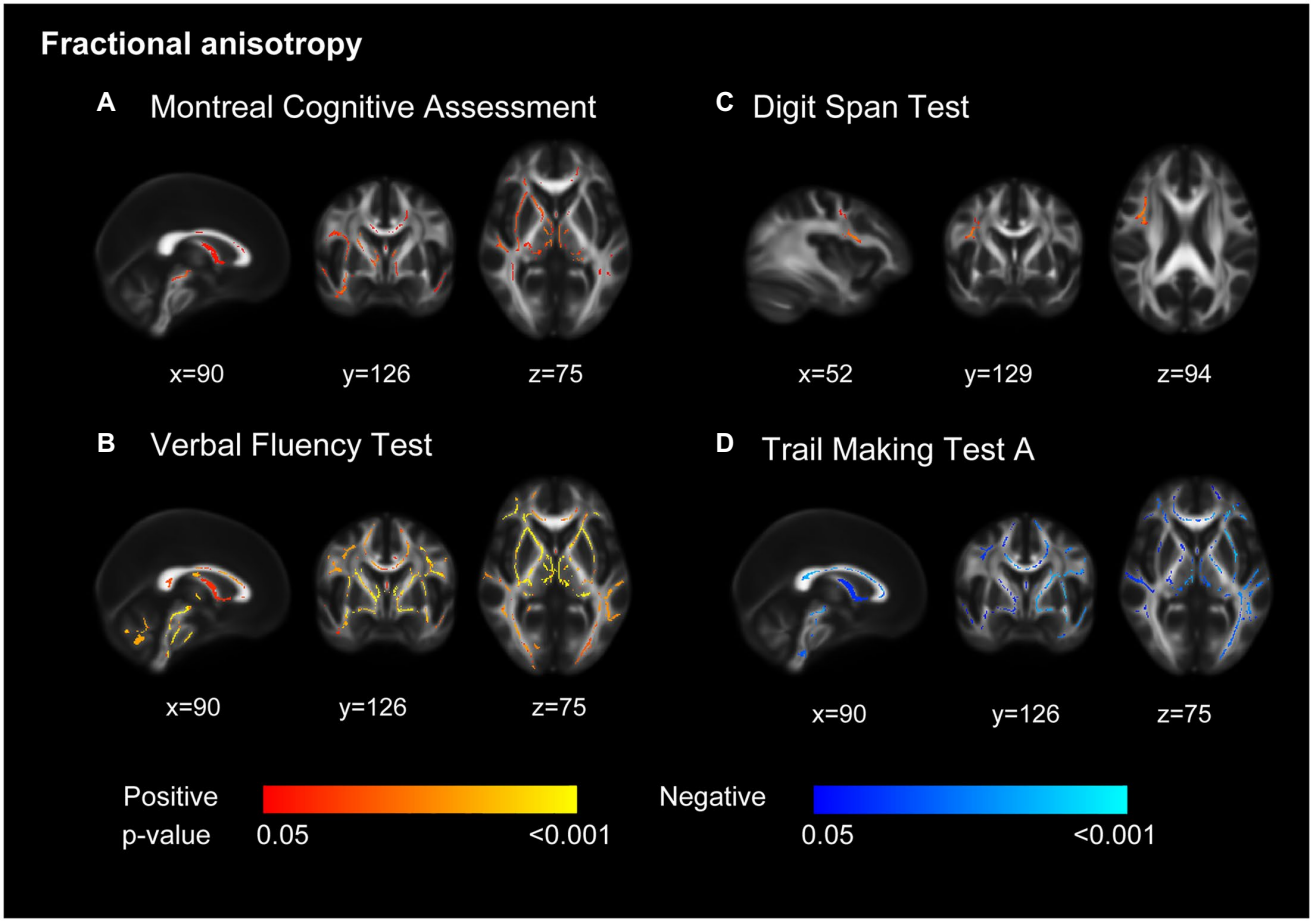
Decreased white matter integrity related to cognition. Decreased fractional anisotropy was associated with lower scores in Montreal Cognitive Assessment (MoCA) **(A)**, Verbal Fluency Test **(B)**, Digit Span Test **(C)**, and longer completion time in Trail Making Test part A **(D)**. Models adjusted for age, sex, education, and ApoE ε4 carrier. All results were significant at *p* < 0.05 (threshold-free cluster enhancement corrected) and overlaid on mean fractional anisotropy map in Montreal Neurological Institute normalized space. The orange and blue lines indicate positive and negative associations between fractional anisotropy and cognition tests.

In the TBSS analysis, we found better performances in VFT and TMT-A (shorter TMT-A completion time) were significantly correlated with increased FA and decreased MD in a wide range of voxels (Figure 1; Supplementary Figure 3). A higher MoCA score was correlated with increased FA, predominantly in the anterior part of the brain. The association of DST score with FA and MD showed a different spatial pattern, which concentrated on regional right frontal white matter tracts. Performances in MMSE and TMT-B were not associated with disruptive WM integrity.

### 3.4. Regional cortical structure related to cognition

**Figure 2:**
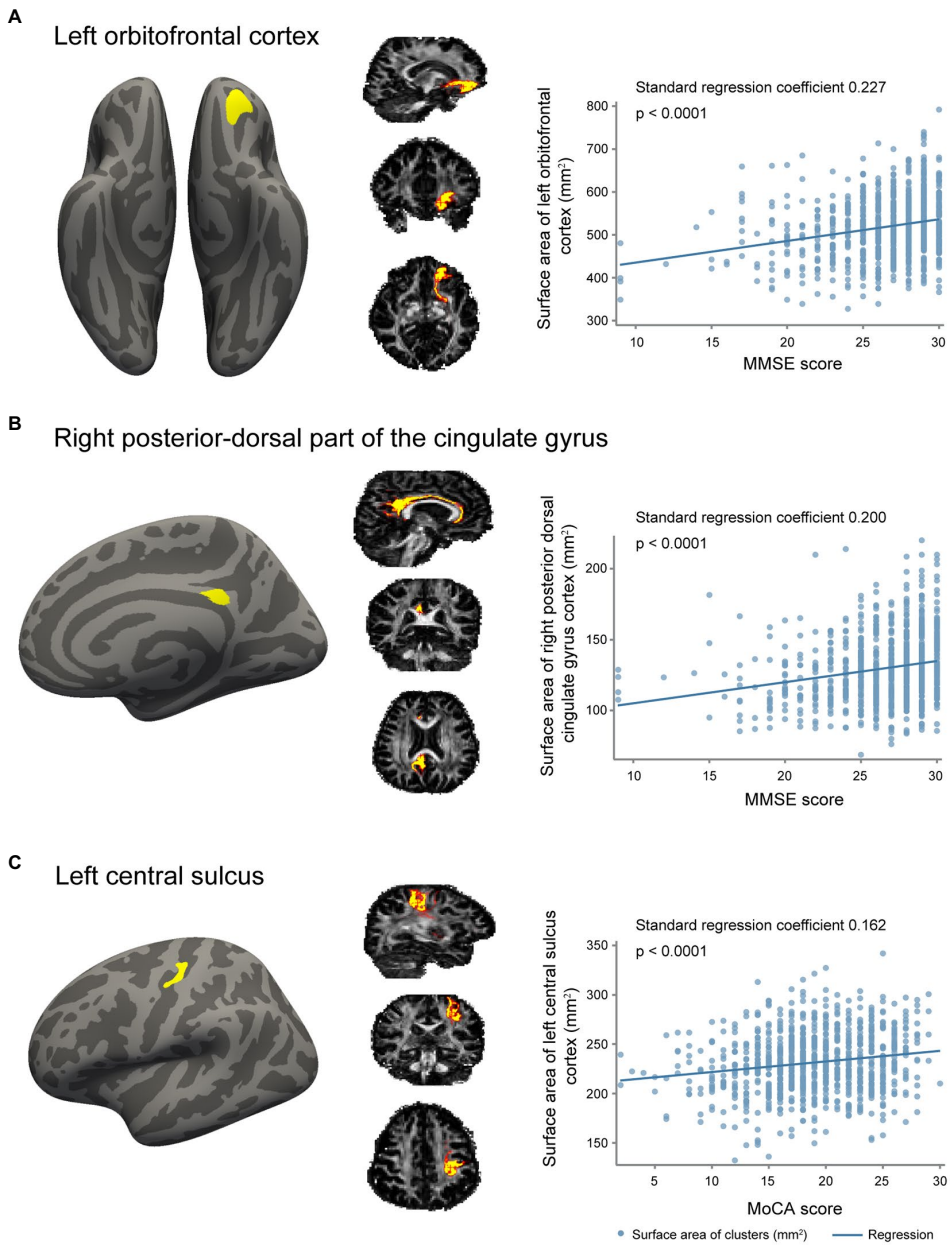
Association of gray matter surface area with cognition. **(A)** The cluster in left orbitofrontal cortex with a surface area positively associated with Mini-Mental State Examination (MMSE) scores, probabilistic tractography of white matter fibers connecting to the cluster, and a scatter plot revealing the association between surface area of this cluster and MMSE scores. **(B)** The cluster in right posterior-dorsal part of the cingulate gyrus with a surface area positively related with MMSE scores, probabilistic tractography of white matter fibers connecting to the cluster, and a scatter plot revealing the association between surface area of this cluster and MMSE scores. **(C)** The cluster in left central sulcus with a surface area positively related with Montreal Cognitive Assessment (MoCA) scores, probabilistic tractography of white matter fibers connecting to the cluster, and a scatter plot revealing the association between surface area of this cluster and MoCA scores.

In the whole brain vertex-wise analysis, we found that the surface area of three clusters was related to cognition (Figure 2). Cortical surface area in the left orbitofrontal cortex and the right posterior-dorsal part of the cingulate gyrus was positively related to MMSE score. Cortical surface area in the left central sulcus was positively associated with MoCA score. We extracted regional surface area of the three clusters. Analysis of the three clusters of interest verified the significant association between surface area and cognition score (Figure 2; Table 3).

**Table 3:** Association of surface area in three clusters with MMSE and MoCA.

	MMSE (Orbitofrontal cortex cluster)			MMSE (Cingulate gyrus cluster)			MoCA (Central sulcus cluster)		
Variable	β	SE	*p*	β	SE	p	β	SE	*p*
**Model 1**									
Cluster surface area	0.227	0.031	<0.001	0.200	0.031	<0.001	0.162	0.031	<0.001
**Model 2**									
Cluster surface area	0.153	0.032	<0.001	0.115	0.029	<0.001	0.164	0.028	<0.001
**Model 3**									
Cluster surface area^a^	0.158	0.033	<0.001	0.118	0.030	<0.001	0.164	0.028	<0.001
Cluster surface area^b^	0.158	0.033	<0.001	0.117	0.030	<0.001	0.165	0.028	<0.001

MMSE indicates Mini-Mental State Examination; MoCA indicates Montreal Cognitive Assessment; β indicates standardized regression coefficient; SE indicates standard error. Model 1: univariate regression. Model 2: adjusted for age, sex, education, ApoE ε4 carrier, and total intracranial volume. Model 3: adjusted for age, sex, education, ApoE ε4 carrier, total intracranial volume, and mean fractional anisotropy^a^ or mean diffusivity^b^ of connecting white matter tract.

To examine whether the relationship between cortical surface area and cognition was influenced by its connecting white matter tract, we additionally corrected for the mean FA or MD of white matter tracts connecting to the significant clusters. We found that the associations between surface area and cognition were independent of the connecting white matter tract (Table 3 model 3).

In the sensitivity analyses, similar results were yielded after excluding 50 participants with lower MMSE scores (Supplementary Tables 2, 3).

## 4. Discussion

In this population-based sample, we investigated global and regional changes in white matter and cortical structures related to cognitive function. We found that disruptive white matter integrity was significantly related to cognition impairment, mainly in visuomotor processing speed, semantic memory, and executive function (TMT-A and VFT), rather than verbal short-term memory and working memory (DST). In addition, regional reduced surface area in the left orbitofrontal cortex, right posterior-dorsal part of the cingulate gyrus and left central sulcus was also associated with global cognition, and this association was independent of their connecting white matter tract. These results suggest that both widespread white matter injury and decreased surface area of gray matter in specific regions play roles in cognition.

In our previous study (Wang et al., 2021), we found that reduced subcortical white matter fraction was the major contributor to the worse global cognition. In extension to this study, we used DTI to measure the white matter microstructure and investigated the topological changes of white matter related to cognition in the present study. The results demonstrated that widespread white matter impairment was associated with poor performance in MoCA, VFT, and TMT-A. Regional white matter injury in the right frontal lobe was associated with lower scores in DST. Disrupted white matter microstructures and connectivity networks were common in cerebral small vessel disease (Ter Telgte et al., 2018). These parameters were shown to be associated with cognitive impairment both in patients with cerebral small vessel disease and in community-dwelling populations (Lawrence et al., 2014; van Uden et al., 2015; Cremers et al., 2016; Tuladhar et al., 2016; Power et al., 2019). The impairment of white matter integrity might lead to decreased information transfer between different cortical regions and cause cognitive decline.

Interestingly, we also found the regional reduced surface area in the left orbitofrontal cortex, right posterior-dorsal part of the cingulate gyrus and left central sulcus was associated with global cognition (MMSE and MoCA). However, our previous study (Celle et al., 2021) did not find the relationships between cortical measures and cognition because we only chose global cortical gray matter measures in the previous study, and these measures were not sensitive enough to detect the subtle and regional changes in normal populations. Furthermore, we found only surface area, rather than surface thickness and volume, was related to cognition, and these associations were not influenced by the connecting white matter tract. These results indicated that the reduced surface area was not secondary to the disruption of connecting fiber tracts. Similar to our findings, Cox et al. revealed that frontal, temporal, and parietal surface area, instead of thickness, was associated with cognitive aging in community-dwelling older people (Cox et al., 2018). A stronger association between cortical surface area and general cognitive ability was also found in a sample of children and the authors also found cortical area change trajectories in higher and lower cognitive ability groups were parallel through life (Walhovd et al., 2016). These evidences might suggest that the observed relations between regional surface area and cognition could be influenced by genetic variants or early life factors, instead of secondary degeneration of white matter tracts. Orbitofrontal cortex plays important roles in cognition, particularly in executive function (Jonker et al., 2015; Zhou et al., 2021) and the shape of the central sulcus has been revealed to be a marker of motor reserve in Cerebral Autosomal Dominant Arteriopathy with Subcortical Infarcts and Leukoencephalopathy (Jouvent et al., 2016). Atrophy of posterior cingulate cortex was also reported to be related with cognitive impairments (Choo et al., 2010). The surface area of these three clusters was positively associated with global cognition in our analysis. However, only small clusters were detected and caution is warranted in drawing inferences about this relationship. Additional studies are needed to validate these findings.

In the TBSS analysis, we did not find associations between FA or MD alteration with MMSE. The possible explanation was that MMSE was not sensitive enough to detect cognitive impairment (Jia et al., 2021). As discussed above, the relationship of orbitofrontal cortex with MMSE may be caused by genetic or early life factors, instead of aging or secondary degeneration of white matter tract. Therefore, the associations of MMSE with white matter and cortical surface area were not mutually exclusive. The disrupted white matter integrity was mainly associated with visuomotor processing speed, semantic memory, and executive function (TMT-A and VFT), but not with verbal short-term memory and working memory (DST). These were consistent with cognitive decline related to cerebral small vessel disease, which primarily affects the frontal-executive domain (Zanon Zotin et al., 2021). Hippocampus volume was not related to cognitive score in the community-dwelling population.

This study was conducted in a community-based sample. The standard cognitive battery and MRI acquisitions allowed a detailed and valid assessment of cognition and brain structure. There are also some limitations to be addressed. The main limitation of the study is the cross-sectional design. Our findings were not able to determine the cause-effect relationship between neuroimaging differences and cognitive function. Fortunately, the Shunyi study is an ongoing cohort, and we are collecting the follow-up imaging and cognitive scores, which would validate these findings. Another limitation is the generalization of our findings. The participants were collected from rural areas and most of these participants were low educated. Previous studies showed that cognitive reserve, such as level of education, offers increased protection against age-related brain pathology (Cox et al., 2018). Our observed relationship between brain structure and cognition could not be directly generalized to other populations.

## Data availability statement

The raw data supporting the conclusions of this article will be made available by the authors, without undue reservation.

## Ethics statement

The studies involving human participants were reviewed and approved by the Ethics Committee at Peking Union Medical College Hospital (reference No. B-160). The patients/participants provided their written informed consent to participate in this study.

## Author contributions

S-YZ, L-YC, Z-YJ, and Y-CZ: study design. Y-CZ, MY, JN, L-XZ, FH, F-FZ, JY, and W-XL: data acquisition. FH, F-FZ, JY, and W-XL: data analysis. W-XL and F-FZ: writing of first draft. W-XL, F-FZ, JY, and Y-CZ: revising the manuscript for content. All authors contributed to the article and approved the submitted version.

## Funding

This study was supported by the CAMS Innovation Fund for Medical Sciences (CIFMS2021-I2M-C&T-B-012) and the Strategic Priority Research Program “Biological basis of aging and therapeutic strategies” of the Chinese Academy of Sciences (grant XDB39040300).

## Conflict of interest

The authors declare that the research was conducted in the absence of any commercial or financial relationships that could be construed as a potential conflict of interest.

## Publisher’s note

All claims expressed in this article are solely those of the authors and do not necessarily represent those of their affiliated organizations, or those of the publisher, the editors and the reviewers. Any product that may be evaluated in this article, or claim that may be made by its manufacturer, is not guaranteed or endorsed by the publisher.

## Supplementary material

The Supplementary material for this article can be found online at: https://www.frontiersin.org/articles/10.3389/fnagi.2023.1065245/full#supplementary-material

## Footnotes

^1^ https://git.fmrib.ox.ac.uk/falmagro/UK_biobank_pipeline_v_1

^2^ http://surfer.nmr.mgh.harvard.edu/

^3^ http://enigma.ini.usc.edu/protocols/imaging-protocols

^4^ https://surfer.nmr.mgh.harvard.edu/fswiki/FsTutorial/TroubleshootingData

^5^ https://git.fmrib.ox.ac.uk/falmagro/UK_biobank_pipeline_v_1/-/tree/master/bb_diffusion_pipeline

^6^ http://www.psmd-marker.com/

^7^ https://fsl.fmrib.ox.ac.uk/fsl/fslwiki/PALM
