# Supplementary material for: White matter and gray matter changes related to cognition in community populations
Source: Front Aging Neurosci. 2023 Mar 14;15:1065245. doi: 10.3389/fnagi.2023.1065245 (PMC10036909; doi:10.3389/fnagi.2023.1065245)
Supplement: Supplementary file 1 [file Data_Sheet_1.docx]

Supplementary Material


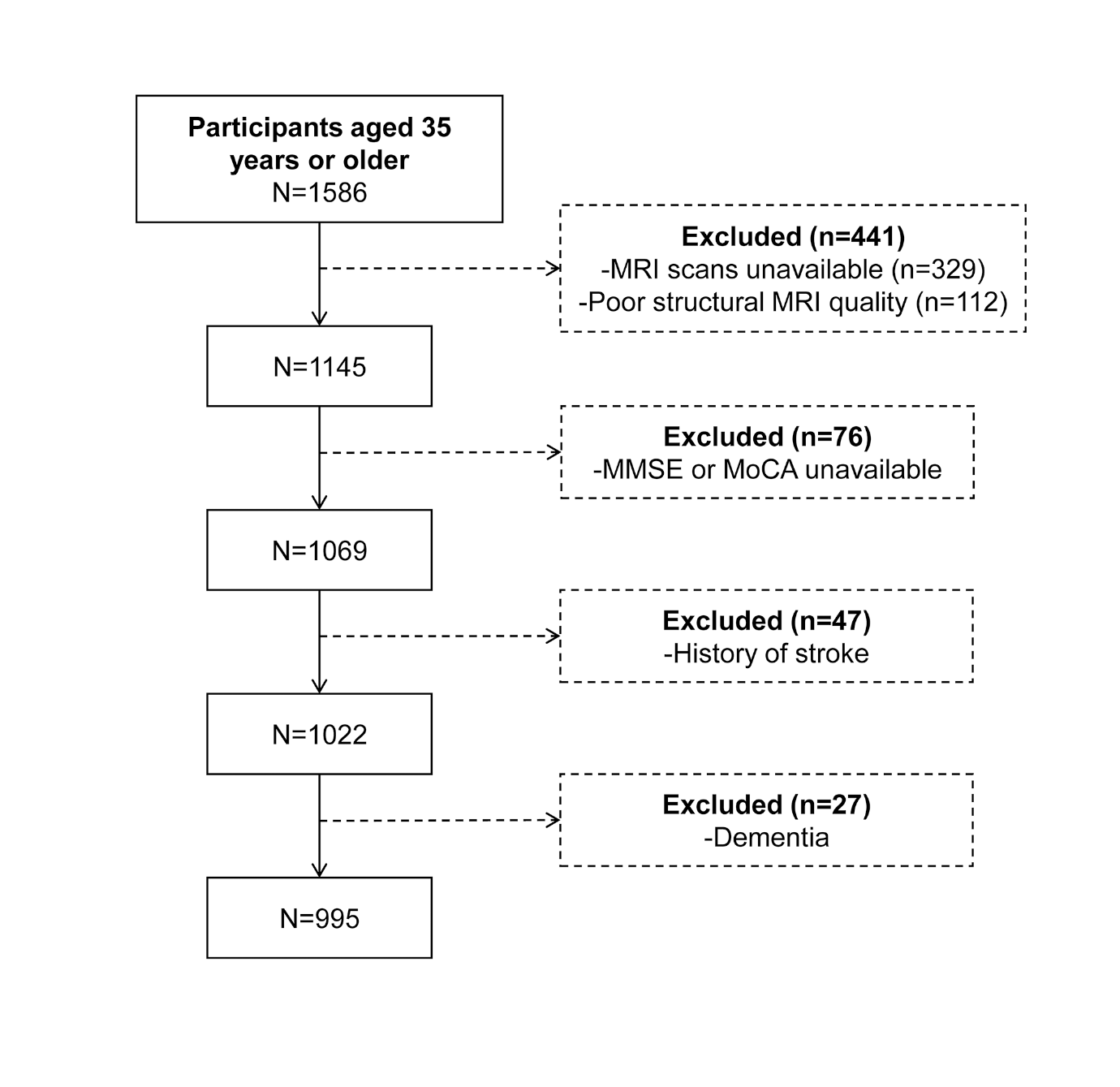


**eFigure 1. Flow chart of the study population.** MRI indicates magnetic resonance imaging.


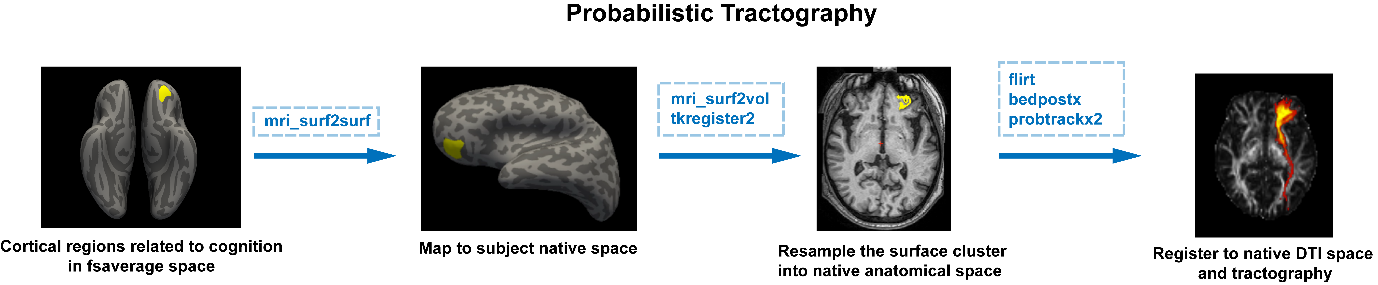


**eFigure 2. Process of probabilistic tractography.**

Cortical clusters related to cognition were extracted from Freesurfer data as the seeding regions. The transformation matrix from Freesurfer surface space to native DTI volume space was constructed via mri_surf2surf, mri_surf2vol, tkregister2, and FLIRT. Probabilistic tractography was conducted via bedpostx and probtractx2 tools.


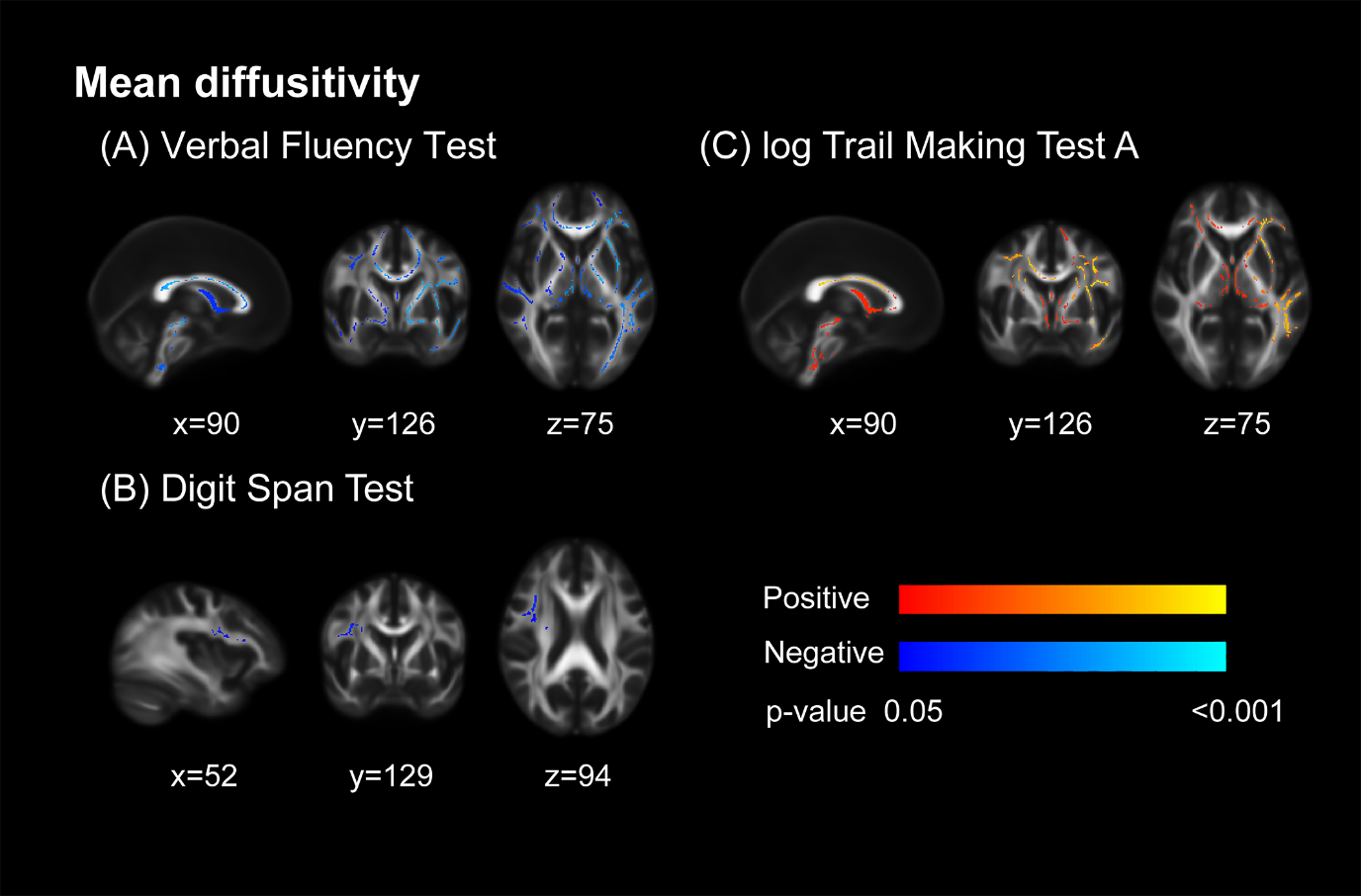


**eFigure 3. Decreased white matter integrity related to cognition (MD map).**

Increased mean diffusivity was associated with lower scores in Verbal Fluency Test (A), Digit Span Test (B), and longer completion time in Trail Making Test part A (C). Models adjusted for age, sex, education, and ApoE ε4 carrier. All results were significant at p<0.05 (threshold-free cluster enhancement corrected) and overlaid on mean fractional anisotropy map in Montreal Neurological Institute normalized space. The orange and blue lines indicate positive and negative associations between fractional anisotropy and cognition tests.

**eTable 1. Univariate analysis of demographics, brain structures and cognition.**

|  | MMSE | | |  | MoCA | | |  | Verbal Fluency Test | | |  | Digit Span Test | | |  | TMT-A | | |  | TMT-B | | |
| --- | --- | --- | --- | --- | --- | --- | --- | --- | --- | --- | --- | --- | --- | --- | --- | --- | --- | --- | --- | --- | --- | --- | --- |
| Variable | β | SE | p |  | β | SE | p |  | β | SE | p |  | β | SE | p |  | β | SE | p |  | β | SE | p |
| Age at MRI, year | -0.39 | 0.03 | **<.001** |  | -0.47 | 0.03 | **<.001** |  | -0.33 | 0.03 | **<.001** |  | -0.26 | 0.03 | **<.001** |  | 0.46 | 0.03 | **<.001** |  | 0.43 | 0.03 | **<.001** |
| Male | 0.23 | 0.07 | **<.001** |  | 0.29 | 0.07 | **<.001** |  | -0.16 | 0.07 | 0.018 |  | 0.16 | 0.07 | 0.018 |  | -0.18 | 0.07 | 0.006 |  | 0.03 | 0.07 | 0.678 |
| Education, year | 0.51 | 0.03 | **<.001** |  | 0.51 | 0.03 | **<.001** |  | 0.34 | 0.03 | **<.001** |  | 0.36 | 0.03 | **<.001** |  | -0.35 | 0.03 | **<.001** |  | -0.31 | 0.03 | **<.001** |
| ApoE ε4 Carrier | 0.07 | 0.09 | 0.450 |  | 0.04 | 0.09 | 0.645 |  | 0.01 | 0.09 | 0.955 |  | 0.10 | 0.09 | 0.260 |  | 0.02 | 0.09 | 0.822 |  | 0.02 | 0.09 | 0.799 |
| Cortical GM volume, mm^3^ | 0.26 | 0.03 | **<.001** |  | 0.29 | 0.03 | **<.001** |  | 0.12 | 0.03 | **<.001** |  | 0.17 | 0.03 | **<.001** |  | -0.23 | 0.03 | **<.001** |  | -0.15 | 0.03 | **<.001** |
| Cortical GM surface area, mm^2^ | 0.23 | 0.03 | **<.001** |  | 0.26 | 0.03 | **<.001** |  | 0.09 | 0.03 | 0.004 |  | 0.16 | 0.03 | **<.001** |  | -0.20 | 0.03 | **<.001** |  | -0.12 | 0.03 | **<.001** |
| Cortical GM thickness, mm | 0.11 | 0.03 | **<.001** |  | 0.12 | 0.03 | **<.001** |  | 0.11 | 0.03 | **<.001** |  | 0.05 | 0.03 | 0.095 |  | -0.10 | 0.03 | 0.002 |  | -0.10 | 0.03 | 0.001 |
| Hippocampus volume, mm^3^ | 0.29 | 0.03 | **<.001** |  | 0.33 | 0.03 | **<.001** |  | 0.17 | 0.03 | **<.001** |  | 0.15 | 0.03 | **<.001** |  | -0.25 | 0.03 | **<.001** |  | -0.18 | 0.03 | **<.001** |
| WMH volume,log | -0.26 | 0.04 | **<.001** |  | -0.36 | 0.04 | **<.001** |  | -0.31 | 0.04 | **<.001** |  | -0.23 | 0.04 | **<.001** |  | 0.38 | 0.04 | **<.001** |  | 0.30 | 0.04 | **<.001** |
| Global FA | 0.20 | 0.03 | **<.001** |  | 0.28 | 0.03 | **<.001** |  | 0.26 | 0.03 | **<.001** |  | 0.17 | 0.03 | **<.001** |  | -0.31 | 0.03 | **<.001** |  | -0.24 | 0.03 | **<.001** |
| Global MD×10^-3^ mm^2^/s | -0.22 | 0.03 | **<.001** |  | -0.27 | 0.03 | **<.001** |  | -0.28 | 0.03 | **<.001** |  | -0.15 | 0.03 | **<.001** |  | 0.33 | 0.03 | **<.001** |  | 0.24 | 0.03 | **<.001** |
| PSMD×10^-3^ mm^2^/s | -0.23 | 0.03 | **<.001** |  | -0.27 | 0.03 | **<.001** |  | -0.25 | 0.03 | **<.001** |  | -0.15 | 0.03 | **<.001** |  | 0.31 | 0.03 | **<.001** |  | 0.22 | 0.03 | **<.001** |

Abbreviations: MMSE indicates Mini-Mental State Examination; MoCA indicates Montreal Cognitive Assessment; TMT-A indicates Trail Making Test part A; TMT-B indicates Trail Making Test part B; GM indicates gray matter; FA indicates fractional anisotropy; MD indicates mean diffusivity; PSMD indicates peak width of skeletonized mean diffusivity. β indicates standardized regression coefficient; SE indicates standard error; p indicates p-value.

^a^Models adjusted for age, sex, education, ApoE ε4 carrier, and total intracranial volume.

^b^Models adjusted for age, sex, education, and ApoE ε4 carrier.

**eTable2**. **Association of cortical gray matter, hippocampus, and white matter measures with cognition (sensitivity analysis).**

|  | MMSE | | | |  | MoCA | | | |  | Verbal Fluency Test | | | |  | Digit Span Test | | | |  | TMT A | | | |  | TMT B | | | |
| --- | --- | --- | --- | --- | --- | --- | --- | --- | --- | --- | --- | --- | --- | --- | --- | --- | --- | --- | --- | --- | --- | --- | --- | --- | --- | --- | --- | --- | --- |
| Variable | β | SE | p | FDR-p |  | β | SE | p | FDR-p |  | β | SE | p | FDR-p |  | β | SE | p | FDR-p |  | β | SE | p | FDR-p |  | β | SE | p | FDR-p |
| Cortical GM volume, mm^3^ | 0.052 | 0.053 | 0.330 | 0.546 |  | 0.057 | 0.051 | 0.267 | 0.531 |  | 0.000 | 0.058 | 0.996 | 0.996 |  | 0.018 | 0.059 | 0.760 | 0.869 |  | 0.003 | 0.055 | 0.954 | 0.974 |  | 0.121 | 0.055 | **0.030** | 0.161 |
| Cortical GM surface area, mm^2^ | 0.050 | 0.058 | 0.382 | 0.566 |  | 0.089 | 0.056 | 0.113 | 0.352 |  | 0.048 | 0.064 | 0.454 | 0.605 |  | 0.052 | 0.064 | 0.418 | 0.589 |  | 0.010 | 0.059 | 0.871 | 0.950 |  | 0.101 | 0.060 | 0.094 | 0.323 |
| Cortical GM thickness, mm | 0.008 | 0.031 | 0.798 | 0.890 |  | -.016 | 0.030 | 0.600 | 0.719 |  | -.039 | 0.034 | 0.258 | 0.531 |  | -.024 | 0.034 | 0.479 | 0.622 |  | 0.004 | 0.032 | 0.895 | 0.955 |  | 0.045 | 0.032 | 0.160 | 0.405 |
| Hippocampus volume, mm^3^ | 0.089 | 0.037 | **0.016** | 0.125 |  | 0.078 | 0.036 | **0.030** | 0.161 |  | 0.049 | 0.041 | 0.230 | 0.525 |  | -.023 | 0.041 | 0.581 | 0.715 |  | -.012 | 0.038 | 0.744 | 0.869 |  | 0.061 | 0.039 | 0.117 | 0.352 |
| WMH volume,log | 0.004 | 0.045 | 0.936 | 0.974 |  | -.050 | 0.044 | 0.254 | 0.531 |  | -.090 | 0.050 | 0.072 | 0.288 |  | -.106 | 0.051 | **0.037** | 0.176 |  | 0.041 | 0.047 | 0.378 | 0.566 |  | -.030 | 0.048 | 0.530 | 0.669 |
| Global FA | 0.037 | 0.034 | 0.277 | 0.531 |  | 0.082 | 0.033 | **0.012** | 0.125 |  | 0.108 | 0.037 | **0.004** | 0.092 |  | 0.068 | 0.038 | 0.072 | 0.288 |  | -.086 | 0.035 | **0.013** | 0.125 |  | -.033 | 0.036 | 0.359 | 0.566 |
| Global MD×10^-3^ mm^2^/s | -.055 | 0.037 | 0.141 | 0.383 |  | -.036 | 0.036 | 0.315 | 0.540 |  | -.124 | 0.041 | **0.002** | 0.092 |  | -.042 | 0.041 | 0.310 | 0.540 |  | 0.094 | 0.038 | **0.014** | 0.125 |  | -.031 | 0.039 | 0.430 | 0.589 |
| PSMD×10^-3^ mm^2^/s | -.037 | 0.037 | 0.311 | 0.540 |  | -.048 | 0.036 | 0.185 | 0.443 |  | -.070 | 0.041 | 0.088 | 0.323 |  | -.035 | 0.041 | 0.389 | 0.566 |  | 0.082 | 0.038 | **0.030** | 0.161 |  | -.057 | 0.039 | 0.144 | 0.383 |

Abbreviations: MMSE indicates Mini-Mental State Examination; MoCA indicates Montreal Cognitive Assessment; TMT-A indicates Trail Making Test part A; TMT-B indicates Trail Making Test part B; GM indicates gray matter; FA indicates fractional anisotropy; MD indicates mean diffusivity; PSMD indicates peak width of skeletonized mean diffusivity. β indicates standardized regression coefficient; SE indicates standard error; FDR-p indicates false discovery rate adjusted p-value.

^a^Models adjusted for age, sex, education, ApoE ε4 carrier, and total intracranial volume.

^b^Models adjusted for age, sex, education, and ApoE ε4 carrier.

**eTable 3**. **Association of surface area in three clusters with MMSE and MoCA (sensitivity analysis).**

|  | MMSE  (Orbitofrontal cortex cluster) | | |  | MMSE  (Cingulate gyrus cluster) | | |  | MoCA  (Central sulcus cluster) | | |
| --- | --- | --- | --- | --- | --- | --- | --- | --- | --- | --- | --- |
| Variable | β | SE | p |  | β | SE | p |  | β | SE | p |
| **Model 1** |  |  |  |  |  |  |  |  |  |  |  |
| Cluster surface area | 0.230 | 0.034 | <.001 |  | 0.197 | 0.034 | <.001 |  | 0.161 | 0.033 | <.001 |
| **Model 2** |  |  |  |  |  |  |  |  |  |  |  |
| Cluster surface area | 0.178 | 0.035 | <.001 |  | 0.124 | 0.033 | <.001 |  | 0.173 | 0.030 | <.001 |
| **Model 3** |  |  |  |  |  |  |  |  |  |  |  |
| Cluster surface area^a^ | 0.177 | 0.035 | <.001 |  | 0.124 | 0.033 | <.001 |  | 0.172 | 0.030 | <.001 |
| Cluster surface area^b^ | 0.178 | 0.035 | <.001 |  | 0.123 | 0.033 | <.001 |  | 0.173 | 0.030 | <.001 |

Abbreviations: MMSE indicates Mini-Mental State Examination; MoCA indicates Montreal Cognitive Assessment. β indicates standardized regression coefficient; SE indicates standard error; p indicates p-value.

^a^Models adjusted for age, sex, education, ApoE ε4 carrier, and total intracranial volume.

^b^Models adjusted for age, sex, education, and ApoE ε4 carrier.
